# Gonadotropin-Releasing Hormone (GnRH) Agonist Implants for Male Dog Fertility Suppression: A Review of Mode of Action, Efficacy, Safety, and Uses

**DOI:** 10.3389/fvets.2020.00483

**Published:** 2020-08-14

**Authors:** Marc Antoine Driancourt, Joyce R. Briggs

**Affiliations:** ^1^Astek Consulting, Chateauneuf, France; ^2^Alliance for Contraception in Cats and Dogs, Portland, OR, United States

**Keywords:** male dog, sterilize, castrate, neuter, contracept, fertility control, chemical castration, non-surgical

## Abstract

At present, only surgical sterilization is available for veterinarians and pet owners seeking suppression of fertility in male dogs, in most countries. An alternative contraceptive alternative approach is GnRH releasing implants that desensitize the pituitary to the stimulatory effects of GnRH and thereby block testicular function (testosterone and sperm production). Two GnRH agonists (deslorelin and azagly-nafarelin) have been researched in controlled release formulations for this purpose. A deslorelin-releasing biodegradable implant, marketed under the name Suprelorin®, has been available in Australia and New Zealand since 2007, the European Union (EU) since 2008, and received regulatory approval in China and Mexico in late 2019. Two versions of the implant are available, one labeled for a minimum of 6 months of fertility suppression in male dogs, and the other for a minimum of 12 months in male dogs. Another GnRH agonist (azagly-nafarelin) was also included in a solid implant (Gonazon®). Research results showed it delivered 6-months to 1 year of suppressed fertility; however, it is not commercialized. This review paper summarizes research on the mechanism of action for these technologies and compiles and interprets the research on efficacy and safety. New findings on usage of the deslorelin releasing implant in countries where veterinarians and pet owners have this option is shared. Research on off-label use of the product in male dogs is also reviewed. This review aims to aid in the evaluation of the deslorelin releasing implant as an adjunct or alternative for surgical sterilization of male dogs.

## Introduction

Veterinarians, pet owners, animal welfare experts, and shelter practitioners seek to suppress fertility and/or reduce testosterone in male dogs for a variety of reasons, including avoiding siring unwanted litters, improving health and welfare, and reducing testosterone influenced behavior. The priority of these reasons for fertility control may differ according to the target population of dogs. Safety and efficacy are paramount for all cohorts. However, the relevance of factors such as duration of treatment, cost profile, accessibility, and administration process will all vary based on the target population, be it pets who are secure in a home, dogs in breeding programs, homeless animals in an animal shelter, or free-roaming or community dogs.

Despite this variety of reasons for treatment, for decades, the standard and often sole means of managing reproduction in male dogs has been surgical castration, also known as neutering. As a result, whereas there are presently multiple pharmaceutical options to treat many veterinary conditions, no pharmaceutical alternatives with regulatory approval are currently available besides the technology discussed in this review. A chemosterilant utilizing zinc gluconate neutralized by arginine and administered via intratesticular injection was approved in the United States in 2003. The product was unsuccessfully marketed from 2003 to 2005 and again from 2014 to 2016. That formula has been also approved in five other counties but is no longer on the market (1). Calcium Chloride dihydrate is a similar formula available via veterinary compounding only, also administered via injection into the testicles and providing permanent sterilization. Use is extremely limited (2). Since the 1980's, a variety of methods for non-surgical fertility control have been studied (3). Despite significant research in this field, in a wide range of approaches including active immunization against GnRH (4), selective destruction of the GnRH binding cells in the pituitary (5) and GnRH antagonist administration (6) no other alternatives have been successfully developed to date. The field received increased support with the Michelson Prize & Grants' 2008 pledge of $50 million in grants for a permanent non-surgical sterilant for male and female dogs and cats whose work continues as of the date of this publication (https://www.michelsonprizeandgrants.org/about/).

The two types of implants discussed in this review rely on different GnRH agonists and different technologies.

The deslorelin releasing implant (DRI/brand name Suprelorin®) was developed in Australia and is currently marketed by Virbac (Carros, France). There are two versions of this DRI. The 4.7 mg implant, which is labeled to suppress fertility in sexually mature male dogs for a minimum of 6 months, is approved for sale in Australia, the European Union (EU), New Zealand, China, and Mexico. It was launched in Australia and New Zealand in 2007, and in the EU in 2008. Approval in China and Mexico occurred in late 2019, with a launch in 2020 (personal communication, Christelle Demongeot Navarro, 26 February 2020 and 23 June 2020). The 9.4 mg implant, which is labeled to suppress fertility in sexually mature male dogs for a minimum of 12 months, is approved and sold in Australia and the EU (7)^1^. The DRI is placed beneath the skin between the dog's shoulder blades; it is not necessary to prepare the implantation site. The product can be repeatedly dosed to extend the period of fertility suppression. The implant is packed in a single use sterile syringe implant device. As packaged for sale, the product has a 3-year shelf life. The product should be stored in a refrigerator (2–8°C or 36–46°F) and not frozen (11).

The azagly-nafarelin releasing implant (ARI—also called Gonazon®) developed by Intervet (Angers, France) was registered in Europe for a 1-year contraceptive effect in bitches, but was never launched. However, there were relevant studies exploring its potential in male dogs (12, 13) that provide useful data on mode of action, efficacy and safety. It is a silicone based solid implant (14 × 3 × 1 mm) containing 18.5 mg azagly nafarelin that is inserted subcutaneously preferably near the umbilicus.

This review paper begins with a discussion of the mechanism of action of both types of implants, followed by a summary of published data on their use for fertility control in male dogs.

Potential ancillary uses of the DRI in male dogs, as well as additional considerations related to health and behavior, are then discussed. The paper finally explores social considerations of the DRI implant, including veterinary survey data that offer preliminary insight on how and why this treatment is currently used in Europe as a lens into how the veterinary community views this non-surgical contraceptive.

## The Mechanism of Action of GnRH Agonist-Releasing Implants

**Figure 1:**
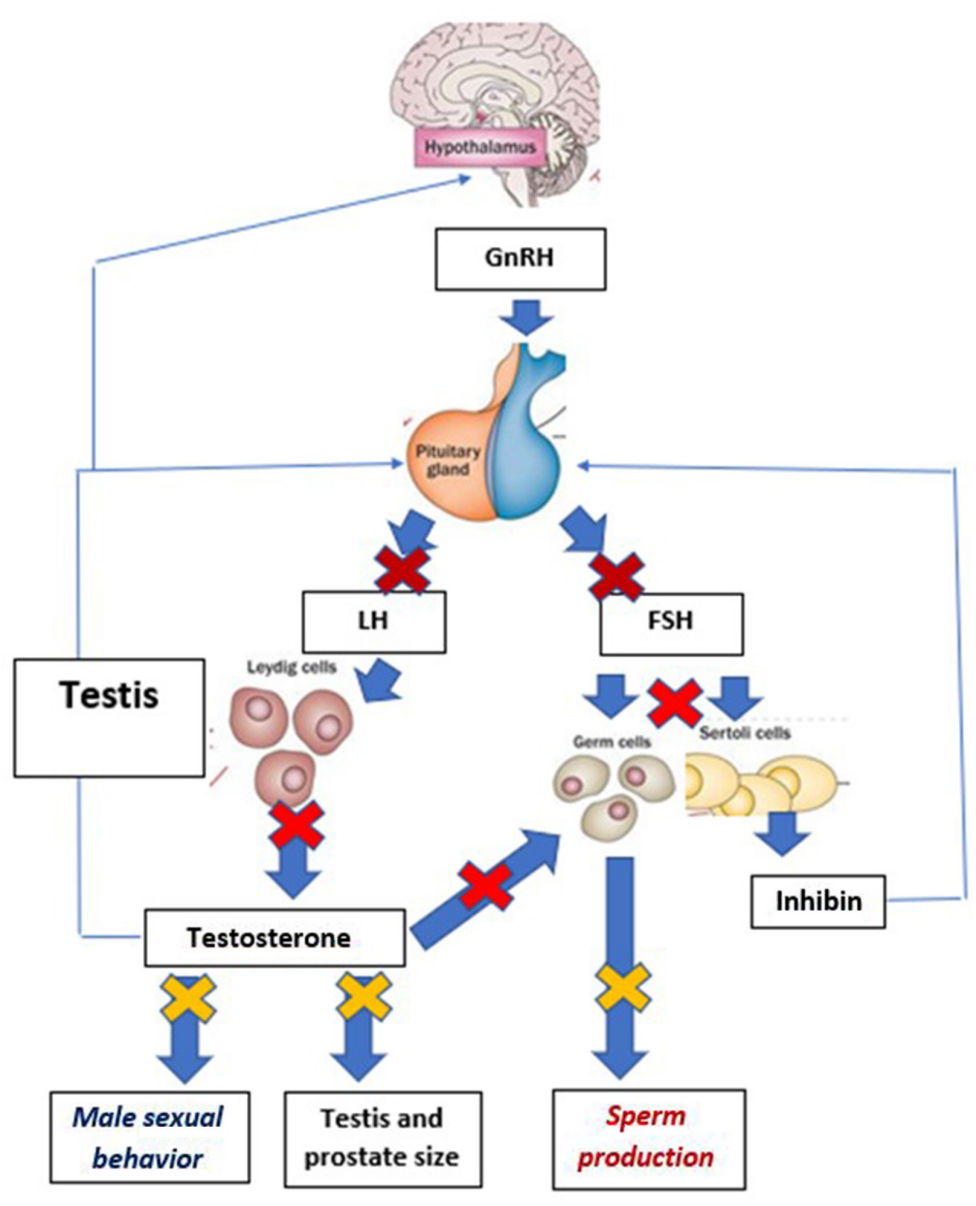
Schematic presentation of the mechanisms involved in the prevention of reproductive function following the desensitization of the pituitary gland to GnRH. Thick arrows demonstrate stimulatory effects, while thin arrows document negative feed-back effects. For clarity of the graph, paracrine, and autocrine regulations within the testis have been omitted. Early effects of desensitization are pictured by 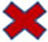. Events occurring later in the cascade of inhibitory effects are shown by 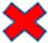 and final ones by 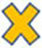. Effects of GnRH desensitization on FSH concentrations in dogs are unknown, as there is no FSH assay validated for dog plasma and therefore no data.

GnRH is a hypothalamic decapeptide, that acts at the top of the cascade that coordinates function of the hypothalamo-pituitary gonadal axis. It is released from specific GnRH-producing neurons in a pulsatile fashion. This pulsatile pattern of release stimulates production and release from the pituitary gland of the two key gonadotropins, follicle stimulating hormone (FSH), and luteinizing hormone (LH), which in turn control gonadal function (in males, testosterone production, and germ cell production, i.e., differentiation of spermatogonia to spermatozoa) (Figure 1).

To date, two GnRH agonists have been used in dogs to alter testicular function: deslorelin (14) and azagly-nafarelin (15). The structures of deslorelin and azagly-nafarelin (vs. the structure of native GnRH) are presented on Table 1. Both GnRH agonists display a peptidic sequence related to the sequence of native GnRH with modifications of three of its amino acids (at positions 1, 6, and 9) and deletion of its amino acid at position 10. The aim of these structural changes is to reduce sensitivity to proteolysis and increase biological activity (16). For example, deslorelin has been found to display a potency 10 or 100 times higher than native GnRH in a GnRH ligand binding test and an *in vitro* culture of rat pituitary cells, respectively (16). There is no similar potency information for azagly-nafarelin.

**Table 1:** Comparative sequences of native GnRH, Deslorelin acetate, and Azagly-nafarelin.

Amino acids 1, 6, and 10 are the ones modified to generates GnRH agonists used in dogs.
Native GnRH: pGlu-His-Trp-Ser-Tyr-Gly-Leu-Arg-Pro-Gly.
Deslorelin acetate: H-Pyr-His-Trp-Ser-Tyr-D-Trp-Leu-Arg-Pro-NHEt.CH_3_CO_2_H.
Azagly-nafarelin: H-Pyr-His-Trp-Ser-Tyr-D-2Nal-Leu-Arg-Pro-NHNHCONH_2._

The purpose of the implant formulations used for dogs is to ensure release of their GnRH agonist content (deslorelin or azagly-nafarelin) for extended durations This was demonstrated in a pharmacokinetic study, published as an abstract (17) describing plasma deslorelin concentrations in dogs following treatment. Plasma deslorelin concentrations peaked ~14 days after insertion of a 4.7 mg implant, reaching concentrations ranging from 200 to 2,000 pg/ ml. Concentrations then gradually decreased and reached undetectable levels at ~80 days post-implantation (17). Comparable data on the 9.4 mg DRI is not published. The release profile of the ARI is not available from the scientific literature. However, an earlier version of the ARI generated release of steady azagly-nafarelin concentrations for up to 350 days (15). The continuous release of either GnRH agonist (deslorelin or azagly-nafarelin) triggers a cascade of biological events (Figure 1).

Continuous exposure of the pituitary cells to GnRH (after a brief stimulatory period, see section Safety and Side Effects GnRH Agonist Implants) triggers pituitary desensitization. Pituitary desensitization to GnRH is triggered by internalization of GnRH receptors (which are no longer present for binding on the cell surface) and inactivation of the intracellular signaling cascade. Once desensitization of the pituitary cells to GnRH is initiated, LH concentrations fall to undetectable values, and therefore fail to support testosterone and sperm production. Such effects have clearly been demonstrated in male dogs (see below and Figure 1). In contrast, owing to the lack of validated FSH assay for dogs, the effects of either type of treatment on FSH secretion have not been established.

The downstream effects of the low gonadotropin concentrations associated with pituitary desensitization are presented in the next section.

## Data on GnRH Agonists Releasing Implants Use in Male Dogs

The suppression of LH and testosterone by the DRI and its effects on sperm numbers and morphology resulting in suppression of fertility were established in multiple studies either using early implant formulations (3, 6, or 12 mg deslorelin) (18–20) or final formulations (14, 21, 22), as well as clinical studies conducted as part of the regulatory approval process of 4.7 and 9.4 mg doses of Suprelorin.

The regulatory studies leading to approval of the 4.7 mg implant included dogs ranging from 10 to 40 kg (22–88 lbs) (14). Regulatory materials submitted to the European Medicines Agency advise that use in dogs outside this range should be subject to a risk/benefit assessment by the veterinarian. Other studies, did, however, test the DRI in dogs under 10 kg (14).

While the ARI implant was developed for contraception in bitches, two detailed studies were conducted in male dogs (12, 13). The combination of all studies with either implant allows comparisons between their respective clinical effects. In addition, some specific aspects (for example reversibility of suppression) could be better studied after the removal of the solid ARI than with the biodegradable DRI (that cannot be easily removed after 6 or 12 months *in situ*).

### Efficacy and Duration of Effect

Measures of efficacy discussed in this section include time to onset of any reduction in testosterone concentrations, time until the dog can be considered contracepted and duration of contraceptive effect. Variability in results are also discussed.

For both ARI and DRI, Table 2 provides a summary of the cascade of events associated/causing prevention of testicular function. It provides evidence that there is a close coincidence between the time when testosterone concentrations reach undetectable values (0.1 ng/ml in most assays) and the time when semen displays features (total sperm numbers, motility, % of abnormal forms) incompatible with fertilization of a bitch (21).

**Table 2:** Changes in relevant endpoints occurring after treatment with a deslorelin releasing implant (DRI) and azagly-nafarelin releasing implant (ARI) and comparison in measurements.

**Implant**	**Deslorelin releasing implant (DRI)**		**Azagly nafarelin releasing implant (ARI)**	
**References**	**Trigg et al**. **(****14****)**^****a****^**;** ***N*** **=** **56 Romagnoli et al**. **(****21****)**^****b****^**;** ***N*** **=** **6**		**Ludwig et al**. **(****12****);** ***N*** **=** **8**	
**End point and metric**	**Time to first reduction (d)**	**Time to full reduction (d)**	**Time to first reduction (w)**	**Time to full reduction (w)**
LH concentrations	ND	ND	ND	Week 11^c^
Testosterone concentrations	Day 23–32^a^	Day 6–43^a^ Day 64–75^a^	Week 1	Week 2–3
Testis size	ND	ND	Week 3	Week 17 (by 80%)
Prostatic size	ND	ND	Week 3	Week 5 (by 46%)
Erection allowing sperm collection	Day 22^b^	Day 30–35^b^	Week 3	Week 5 (semen from 1/8 dogs could be collected)
Sperm volume	Day 37–47^b^	Day 64–75^b^	Week 3 (by 70%)	No sperm collected in 7/8 dogs
Total sperm number in the ejaculate	Day 37–47^b^	Day 64–75^b^	Week 3 (by 55%)	No sperm collected in 7/8 dogs
Sperm motility	Day 23–32^b^	Day 64–75^b^	ND	No sperm collected in 7/8 dogs
% of Abnormal sperm	ND^a^	Increased to 70% by Day 35^a^	Unchanged (at 30%) on week 3	No sperm collected in 7/8 dogs

*Note that the durations are expressed in days for the DRI studies vs. in weeks for the ARI studies. ND, not documented*.

a *Data from Trigg et al. (14), N = 56*.

b *Romagnoli et al. (21), N = 6*.

c *Week 11 was the first time point when LH concentrations were assessed and only for the ARI*.

The cascade leading to shrinkage of the testicles and prostate followed by the inability to produce sperm during collection attempts and low sperm quality (low total sperm numbers, low motility, and increased proportion of abnormal forms) is presented on Figure 1 and the timings of each of these events on Table 2. Irrespective of the implant used, testicles commonly shrink to <50% of the pre-treatment size (12, 20, 23). The proportion of motile sperm, total sperm numbers, and semen volume fall to very low values, incompatible with fertility (12, 21). Data on abnormal sperm following treatment are mixed with one study failing to find an effect on the percentage of abnormal sperm (21), while another one claiming overall a 10-fold increase in abnormalities of sperm tails (from 7 to 70%) at 35 days after treatment (14).

Lack of libido (likely due to suppression of testosterone production), combined with an arrest of spermatogenesis at the spermatogonia/spermatocyte stage leading to azoospermia were shown as key factors involved in achieving contraception.

The approved label claim for the commercialized DRI is to prevent testicular function and block fertility in male dogs for a minimum of six (4.7 mg) or 12 months (9.4 mg). In 9/10 dogs of each of two dose groups, the 4.7 and 9.4 mg the DRI was shown in registration studies to consistently suppress testosterone (below the assay's limit of quantification, 0.1 ng/ml) for at least 180 and 400 days, respectively (*N* = 10/group) (14).

There are indications of a correlation between a dog's body weight and duration of fertility suppression, with a generally longer period of testosterone suppression observed in small dogs (weighing <10 kg) than in dogs over 10 kg (14). During clinical trials with the 4.7 mg implant, most of the smaller dogs (<10 kg) maintained suppressed levels of testosterone for more than 12 months following implantation (11, 14).

Variability in the duration of fertility suppression is generally large. In dogs ranging in weight from 10 to 25 kg (*n* = 31) and receiving the 4.7 mg deslorelin releasing implant, the duration of undetectable testosterone concentrations (under 0.1 ng/ml) ranged from 150 to 500 days (14). A similar variability was detected in dogs <10 kg (*n* = 11: 200–570 days). Furthermore, following the use of a 9.4 mg deslorelin implant in 10 dogs, undetectable testosterone concentrations ranged from 322 days post-implantation in the dog with the shortest duration, to more than 700 days in the two dogs showing longest response (14).

Duration of efficacy of the ARI was evaluated in two studies (12, 13). In the first one (12), two groups of dogs (*n* = 4/group) received the implant for 6 or 12 months. Undetectable testosterone concentrations (below 0.1 ng/ml) were maintained for the duration of the study for 4/4 dogs of the 6 months group and in 1/4 dogs of the 12 months group. Resumption of testosterone concentrations above 0.5 ng/ml was observed on days 223, 307, and 324 post-insertion in the 3 other dogs. In a second study, suppression of testosterone to undetectable concentrations for 6 months was observed in 46/53 dogs while 5/53 dogs displayed decreased but detectable testosterone concentrations. Failure (no testosterone suppression) was reported in 1/53 dogs (13). There were too few dogs to assess variation by weight category.

Variability in the time required to reach undetectable testosterone concentrations (hence demonstrating pituitary desensitization) is also well-documented (14, 20, 21). In one study in 56 dogs of three different weight groups (below 10 kg, 10–25 kg, and over 25 kg) receiving the 4.7 mg implant, testosterone concentrations reached undetectable values between 6 and 43 days following treatment (14). Romagnoli et al. (21) similarly used a 4.7 mg implant in a study of six dogs of varying weights (7.8–40.2 kg). Sterility (based on semen collection and sperm features in the ejaculate) was detected between 54 ± 21 days post-treatment. Neither of these studies reports a link between body weight of the treated dogs and this variability. Taking into account multiple studies on the subject, it can be stated that the onset of complete suppression of fertility with the 4.7 mg DRI implant may typically occur between 1 and 2 months after treatment. No data on this is available for ARI.

The mechanisms causing this variability are not understood. The links between the actual release profile of deslorelin or azagly-nafarelin by these implants and this variability remain to be explored.

Sexual behavior in males is driven by testosterone. Hence, in addition to having the potential to suppress testosterone and sperm production, blocking the effects of GnRH also reduces or abolishes the physiological response of penile erection and non-habituated behaviors related to sex hormones (3)^2^. Irrespective of the implant used, libido (as measured by thrusting action) was markedly reduced. In a study with the 4.7 mg DRI (*n* = 55), libido which was present pre-treatment in 70% of the 56 treated dogs, had decreased to 50% by Day 22 post-implantation and ceased between 30 and 35 days (14). In another study with the ARI, in which semen collections were attempted every other week, the last ejaculate could be collected during week 3 after treatment in 7/8 dogs and during week 5 in the last dog (12).

### Reversibility and Retreatment

Veterinarians and clients may have different objectives for using GnRH agonists releasing implants. One goal is to achieve ongoing suppression of testicular function and fertility. A different goal may be to achieve temporary suppression of testicular function, to preview the effects of castration, or to delay the decision to breed a male dog. In all these scenarios, it is important to know when to re-implant, castrate or assume a dog can successfully sire a litter.

As the ARI is made of silicone, it can be surgically removed to end treatment and begin the sequence of events leading to resumption of testicular function. Such a sequence and the timing of the consecutive steps are presented in Table 3. Overall, it took around 24–26 weeks for testicles to grow back to their pre-treatment size and 29–30 weeks for “normal” sperm production to occur (12).

**Table 3:** Sequence of events occurring after the end of GnRH desensitization with a Gonazon implant (*N* = 5 dogs) (12).

	**End point**	**Initiation of recovery (w)**	**Full recovery (w)**
Step 1	Resumption of LH secretion^a^	Week 4 post-implant removal	Week 12 post-implant removal
Step 2	Recovery of testosterone concentrations^b^	Week 4 post-implant removal	Week 7 post-implant removal
Step 3	Recovery of testis size^b^	Week 6 post-implant removal	Week 24–26 post-implant removal
Step 4	Recovery of prostate size^b^	Week 8 post-implant removal	Week 16 post-implant removal
Step 5	Recovery of a normal sperm production^c^	ND	Week 29–30 post-implant removal

a *LH secretion post-treatment was only evaluated on two occasions: week 4 and week 12*.

b *Testosterone concentrations together with testicular and prostatic size were assessed weekly till week 12 then monthly (weeks 16, 20, and 24/26)*.

c *Numerical data are only reported for the week 29–30 time window. ND, not determined*.

Studies of DRI demonstrate that testicular function, and semen quality return to normal values after the end of release of the GnRH agonist included in the implant (14), but the timing of resumption of fertility is variable (14, 18).

In contrast to the ARI, the DRI is fully degraded at the end of its release period. To maintain suppression of fertility, the DRI label recommends re-implanting at either 6-month (4.7 mg) or 12-month (9.4 mg) intervals. Data do not further address precise timing of resumption of reproductive function following use of the implant. An owner can monitor the signs of return to fertility in making a reimplantation decision. On the other hand, owners should understand that if they want to breed their dogs after using a DRI, return to fertility may be delayed.

Use of the DRI for long-term contraception therefore requires consecutive treatments. During registration studies, 10 dogs were implanted four consecutive times with a 4.7 mg DRI, and testosterone was monitored during and after treatment. Dogs were observed for any adverse events. The study demonstrated that re-implantation of previously treated dogs is safe and serves to continuously suppress fertility (14). The dossier submitted for regulatory approval in Europe reports safety and efficacy with four and five sequential treatments with the 4.7 mg implant (24). It is assumed the implant can be used for a longer duration but published studies documenting that are not found in the literature.

### Safety and Side Effects GnRH Agonist Implants

Very few adverse effects were recorded during studies done with either type of implant with the exception of moderate swelling at the implant site commonly reported for 14 days for DRI (11), and a short term flare up phase described in this section.

Other side effects during the DRI release period were very uncommon (occurrence below 0.1% of the treated dogs) and limited to coat abnormalities or an owner reported reduction of general activity for some weeks after treatment (11, 22).

For the ARI, a study in 8 male beagles noted no local reactions at the site of implantation; no other adverse reactions were noted throughout the study period (6–12 months) (12). In a study with 53 male dogs being treated with the ARI as an alternative treatment to castration for benign prostatic hyperplasia (BPH) and for behavioral issues, minimal side effects were recorded (13).

During the first days post-implantation, there is a short-lived step during which exposure to the GnRH agonist has stimulatory effects on the pituitary gonadal axis (“flare-up phase”). During this “flare-up phase,” an acute increase in serum testosterone occurs. This was documented in a study involving six dogs treated with the 4.7 mg DRI which identified increased testosterone concentrations between Days 1 and 5 post-treatment (25). In another study (21), a short-lived increase in semen motility was observed on days 9–17 post-implantation of a 4.7 mg DRI (*n* = 6). This flare-up period may cause transitory changes in behavior. In one study involving 24 dogs treated with a 4.7 mg DRI, owners reported an increase in sexual behavior in eight dogs 1 week after treatment, dropping to three dogs by week 3. Owners of five dogs reported increased aggression toward male dogs 1 week following treatment, dropping to two dogs by week 5 (22). Cautions are provided about using DRI in dogs with sociopathic disorders related to aggression (11), in part due to possible heightened behavior response during the flare-up phase.

This flare-up phase ends when desensitization of the pituitary cells to GnRH is initiated, at which point testosterone concentrations start dropping.

## Potential Ancillary Uses of the Deslorelin Releasing Implant in Male Dogs

Research has revealed potential uses of GnRH agonist implants in male dogs beyond providing fertility suppression in sexually mature animals. Three off-label uses of the product are described below. Off-label research in female dogs and other species is beyond the scope of this paper^3^.

### Fertility Control in Prepubertal Male Dogs

In some countries, including Australia and the United States, veterinarian, and/or shelter recommendation of prepubertal sterilization of dogs is relatively common (26, 27). In the United States, it has been recommended that shelters perform neutering prior to adoption, as early as 6 weeks of age (28).

The appeal of surgical sterilization in young dogs in some countries begs the question of whether chemical fertility control in this age cohort is safe and effective. Efficacy in male puppies was documented by a small study in Beagle and mixed breed dogs treated at 4 months of age. Treatment of 4-month-old male pups (*n* = 4) with a 4.7 mg DRI postponed puberty (defined using behavior and testicular size as end points) until 2.5 years of age (29). When a 9.4 mg DRI was used (*n* = 4), puberty failed to be detected before termination of the study, when dogs were between 2.5 and 3.2 years of age. No significant side effects on growth, size, or height were detected in this study (29). Given the limited sample size of this study, it is relevant to also note that in a study of nine prepubertal female dogs treated with DRI of both doses, neither body development (height, humeral length) nor body weight was affected by treatment, despite a significant delay in epiphyseal closure (30). These studies suggest that prepubertal treatment of dogs with GnRH agonist implants may be safely used to postpone puberty in dogs without interfering with their final size or height. No studies have been done with ARI on prepubertal dogs.

### Treatment of Behavioral Problems in Intact Male Dogs

Some intact male dogs exhibit some behaviors that owners find objectionable. Surgical castration is commonly proposed by veterinarians to alleviate such behaviors in some dogs (31). Due to their ability to suppress testosterone, GnRH agonists releasing implants may also be useful for altering/minimizing behaviors related to testosterone. Improvements in sexually dimorphic male behaviors described as libido, hypersexuality, intermale conflict, and excessive territorial urine marking have all been described for DRI (32). The product label of the DRI in Australia specifies that the product can be used as an “aid in the control of unacceptable behavior.”

In a study comparing the effects of surgical castration and treatment with a DRI on male dog behavior (22), 24 intact, sexually mature dogs of varied sizes and breeds received a 4.7 mg implant. They weighed 26.2 (±14.2) kg and were aged 41 (±22.6) months old. None of the dogs entered the study with a specific problem behavior as perceived by their owner. Owners assessed their behavior for seven weeks after treatment. Sexual behavior decreased in a majority of implanted dogs 3 weeks after treatment, and in all but two implanted dogs by 7 weeks (those two dogs' behavior had not changed). Owner reported aggression toward male dogs decreased in 79% of implanted animals by 5 weeks following treatment, and urine marking either remained stable or decreased in this timeframe.

In a study with the ARI (13), involving 26 dogs (age: 0.6–16 years, weight ranging between <10 kg and more than 30 kg) presenting hypersexuality, clinical signs decreased significantly in 24 dogs. Loss of libido was generally detected 8 weeks after implantation. In 19 dogs combining hypersexuality and aggressive behavior, a significant improvement of both conditions was detected in 10 of them as described by their owners (13)^4^.

It appears that GnRH agonist releasing implants are an alternative for those seeking change in testosterone mediated behavior in male dogs, without undergoing castration, or as a trial to what might be achieved by permanent sterilization.

### Treatment of Benign Prostatic Hyperplasia in Intact Male Dogs

Benign prostatic hyperplasia (BPH) is a common pathology of the intact male dog, with 47.5% of old (mean age 7–8.6 years) dogs (*n* = 1,003) displaying abnormalities in prostatic features when examined by ultrasonography (33). As prostatic growth is testosterone-dependent, GnRH agonists can be used to reduce the size of the prostate gland (23) and therefore alleviate clinical signs of BPH. DRI and ARI (13, 25) have been studied for this purpose.

Evaluation of the efficacy of DRI in dogs with clinical symptoms of BPH has been conducted in small studies. In one trial, where eight client-owned dogs diagnosed with BPH were treated with a 4.7 mg deslorelin implant and monitored for 24 weeks, the dogs' prostates shrunk steadily to 75% reduction at 16 weeks (34). A second study in six dogs with BPH using a 4.7 mg deslorelin implant reported a progressive shrinkage of prostate volume starting on day 11, becoming significant on day 37 and reaching minimal values (around 20% of its initial volume) on day 52 post-treatment (25). In another study using the ARI and involving 18 dogs, prostatic size had decreased by 45 and 59% at week 8 and 26 post-treatment, respectively. Clinical signs present at inclusion progressively vanished during the first 8 weeks post-treatment (13). Based on these 3 studies, it may be concluded that GnRH agonist releasing implants are an effective method to treat BPH. It is important to note that initially, due to the “flare-up” effect of deslorelin treatment, it is possible that clinical signs associated with BPH may increase for a short period of time.

## Additional Considerations for Health and Welfare of Male Dogs

Beyond the direct effect of rendering a dog sterile, surgical neutering has been found to decrease the incidence of some health conditions, and increase that of others, as compared to dogs who remain intact. GnRH agonist releasing implants could, in theory, have the potential to impact the probability of certain undesirable conditions (such as obesity and cancer) while still offering contraceptive benefits. Although existing research is insufficient to draw conclusions about the effects of GnRH agonist releasing implants relative to surgical castration or no fertility control, the health conditions noted below are areas warranting further research.

### Obesity

Gonadectomy is associated with higher incidence of obesity in dogs in several studies (35–37), and neutering is generally considered to be a risk factor for weight gain (38).

There is very limited information documenting whether factors contributing to obesity following surgical sterilization also exist following fertility control using GnRH agonists releasing implants. Clinical studies with DRI were not designed to evaluate this outcome, as they were mostly involving small sample sizes (therefore generating a limited statistical power) of breeds with a wide range in body weights. In addition, the limited duration of such studies may have been too short to pick up long-term changes in body weight. Among the limited data that exist, one 5-month study found that 10 out of 14 adult male dogs demonstrated a voluntary increase in food intake following DRI treatment, although changes in weight were not measured (39). Two studies involving prepubertal dogs of each sex treated with DRI did not show notable differences in body weight over periods of 40 weeks (*n* = 4 controls and 9 treated dogs) (30) or 32 months following treatment (*n* = 3 controls and 8 treated dogs) (29) between treatment and control groups. However, sample sizes in these studies are believed to be too small to generate solid information.

The strongest evidence documenting body weight changes following fertility control with GnRH agonists was generated in a study in prepubertal bitches treated with an ARI (40). In this study, 20 Beagle bitches (10 pairs of sisters) were either treated with an ARI left *in situ* for 1 year or a placebo implant, and body weight was measured monthly. The experimental design (using pair of sisters allocated to specific treatments) optimized statistical power. This study demonstrated no increase in body weight in the sisters exposed to the ARI for the 12 months of treatment vs. the control pair. Additionally, in a follow up study of 53 client-owned male dogs receiving an ARI, 50/53 dogs did not demonstrate any increased appetite and weight gain according to their owners (13).

Although data on male dogs and DRI specifically are limited, if confirmed, less likelihood of weight gain might be an attractive feature of the implant rather than traditional neutering. In addition, obesity (if developed following fertility suppression using GnRH agonists) might be reversed by interrupting treatment. Further research in this area is needed both to explore the interaction between age of administration and the effect of treatment on body weight in male dogs, and to directly address a comparison between neutering vs. GnRH agonist implant treatment on body weight.

### Cancer

In one published study comprising over 120,000 canine diagnostic records, prevalence of lymphoma, mast cell tumors, osteosarcoma, and adenoma/adenocarcinoma was increased in neutered male dogs relative to their intact counterparts (41). (It is important to note that this was not a prospective study and correlation does not prove causation).

With lymphoma specifically, the role of increased LH concentrations induced by neutering has been hypothesized to play a role in initiation of cancer because LH receptors have been detected in dog lymphocytes through use of immunohistochemistry and flow cytometry (42). Although additional research is needed to demonstrate that the LH receptors detected on lymphocytes are functional receptors that bind LH and activate the intracellular signalization cascade, treatment with GnRH agonist implants decreases serum LH, in contrast to the increased serum LH levels seen in surgically castrated dogs. Therefore, some have hypothesized that if the LH receptors found on lymphocytes are somehow related to the development of lymphoma, then GnRH agonist use, instead of surgical castration, may reduce the risk of lymphoma or its rate of progression. Research is needed to explore this hypothesis.

## Human Attitudes, Behavior, and Non-Surgical Fertility Control

When focusing on neuter status of companion animals in particular, human behavior is an essential variable. By extension, it warrants consideration when discussing ways GnRH agonist releasing implants can be of value for male dogs.

Drawing from studies conducted in various countries, there are a number of interconnected factors that have been shown to influence individual pet owners' choices regarding surgically neutering their dogs. These include the procedure's effects on an animal's reproductive capability, health, and welfare, and behavior, as well as personal beliefs about the procedure's associated pain or necessity (all of which can generate arguments for or against the procedure); the procedure's cost and accessibility; veterinary advice; and fundamental awareness and knowledge that castration is an option (27, 44–49).

It seems likely that if these factors influence decision-making regarding surgical castration, they could influence decision-making regarding non-surgical contraceptive alternatives, as well.

There are a limited number of studies on human behaviors regarding surgical castration compared to non-surgical fertility control for companion dogs in general (50–52), and fewer still on attitudes and behaviors regarding use of the commercialized DRI, Suprelorin specifically (53, 54). Surveys to date have been conducted among veterinarians. While not the ultimate decision-makers regarding use of a product or procedure, veterinarians are a key influencer of owners' and guardians' decisions around whether and how to control the dog's fertility. Despite their limited breadth, these studies provide insight into the reasons why the DRI is used in European countries and serve as a foundation for envisioning how the product might serve male dogs, as well as their owners and guardians.

In an online survey conducted in the United Kingdom and completed by 411 veterinary surgeons, 62% of respondents reported that they always recommend surgical neutering of male dogs not intended for breeding; a higher percentage (94%) do so for female dogs. Among the veterinarians surveyed 33% named Suprelorin as an alternative they offer to surgical neutering for male dogs (53).

A small online survey focused on Suprelorin was conducted with 14 European general practice veterinarians and reproductive specialists. Given the small sample size, results are anecdotal. Overall, respondents reported that ~60% of their male canine patients are left intact, 25% are castrated, and 15% are treated with Suprelorin. All 14 respondents use Suprelorin in their practices, with the majority having offered it in their practice for more than 5 years.

These veterinarians mainly treated male dogs with the 4.7 mg implant. In order of frequency, the reasons they recommend Suprelorin are:

- When surgery is a risk (age/risk of anesthesia)
- To provide reversible fertility suppression of breeding males
- When owners are reluctant to castrate
- As a “test-run” before surgical castration
- When male and female dogs are living in the same household for a temporary period
- To manage inappropriate behavior
- When owners want to avoid any risk of reproduction
- To treat BPH
- To treat perianal gland tumors.

Some practitioners reported using Suprelorin off-label in dogs and other species. In dogs, off-label uses included treatment of BPH in intact males and Alopecia X or sexual aggression in castrated male dogs, and both estrus suppression in female dogs and estrus induction in breeding bitches.

Respondents in this small survey reported that typically the veterinarian suggests use of Suprelorin more so than clients requesting it. They estimated that roughly 85% of veterinarians and about 50% of dog owners are aware of the product. These same professionals reported that about 80% of their clients are either extremely satisfied or somewhat satisfied with the product (equally divided).

## Implications for Use of the Deslorelin Releasing Implant

When viewing the possibilities of the commercially available DRI as an option for veterinarians and those caring for male dogs, here are a number of factors that are worth considering.

### Dog Health and Behavior

Increasing research is taking place into the health effects of surgical castration. At present, findings are somewhat conflicting in terms of the “cost-benefit” analysis for castration, with variables including but not limited to dog breed, size, and age at the time of the procedure [see (43, 55–57)]. Assessments of behavior, and the effects of neutering on male dog behavior, are similarly complex.

This noted, data suggest that the DRI can have a positive effect on benign prostatic hyperplasia and on some behaviors in male dogs as reviewed in this paper. Equally significant, it offers the benefit of “trialing” the effects of castration on behavior or health considerations of interest to the particular dog owner.

### Cost

The price of a DRI to the client includes the cost of the product itself, the labor cost for the veterinarian to conduct a physical exam on the animal and insert the implant and the mark up on both of these components.

In the small online survey of European veterinarians, there was significant variation in the cost to clients for treatment with the DRI; there was also significant variation in the cost of castration surgery. In general, the cost to the client of implanting a dog twice with the 4.7 mg implant would approach the cost of castration surgery (58).

The DRI is not an inexpensive alternative to surgical sterilization, particularly when repeat dosing is desired for ongoing fertility suppression. Its value may be better realized in other capacities, discussed below.

### Owner Preferences and Access to Care

One of the primary values of the deslorelin releasing implant, in the authors' opinion, is fulfilling owner preferences for veterinary care. Veterinarians' survey responses to why they recommend a DRI suggest that there is some demand for alternatives to surgical castration.

There is value in giving pet owners a way to prevent their dog from reproducing without permanently altering his physical appearance, removing his testicles, or requiring surgery and associated anesthesia risk. In addition, the value of a “test run” prior to surgical castration, as some veterinarians noted, should similarly not be overlooked. Due to the fact that this DRI has the same effects on testosterone as surgical castration for its duration of efficacy, owners can preview whether their dog's behavior would change if he were castrated and make a better informed decision about how to proceed.

Finally, the technology could offer value to dog breeders to postpone breeding until a dog's initial quality as a stud can be assessed, or permit male dogs taking a break from breeding to interact with bitches without concern of unwanted litters and, potentially, undesired behaviors. However, the variability seen among individual animals, combined with general trends of duration of efficacy relative to the dog's size, need to be taken into account when planning for product use and breeding schedules.

## Conclusion

GnRH agonists releasing implants offer a demonstrated safe, effective means of temporary chemical suppression of testosterone and of fertility without surgery. This suppression may be extended if several implants are used sequentially. Some effects of such treatments are similar to surgical castration, and others differ. This technology offers an alternative to meet owners' individual preferences and needs for veterinary care. Further research is warranted on potential health benefits of GnRH agonists releasing implants unrelated to fertility control.

## Author Contributions

JB conceived of the project. The manuscript was researched, written, and edited by MD and JB. All authors read and approved the final manuscript.

## Conflict of Interest

The authors declare that the research was conducted in the absence of any commercial or financial relationships that could be construed as a potential conflict of interest.

## Footnotes

^1^In addition to approval for suppression of fertility in sexually mature male dogs, the 9.4 mg dose of Suprelorin has been approved in the EU and Australia for suppression of fertility in male ferrets (8, 9). In the United States, 4.7 mg Suprelorin® F is a U.S. Food & Drug Administration (FDA) Indexed Product to manage adrenal gland disease in sterilized and sexually intact male and female ferrets (10). It is not FDA approved. Extra-label use of Suprelorin F is prohibited and grounds for removal from the market.

^2^Note, researchers recorded thrusting action as a measure of libido; they did not specify if male dogs were in the presence of an estrous female to invoke the behavior.

^3^A product profile and position statement on Suprelorin in female dogs and other species can be found at: https://acc-d.org/docs/default-source/Resource-Library-Docs/Suprelorin4P.

^4^In this study, researchers described the term hypersexuality as comprised of excessive mounting, whining, refusal of food uptake due to the presence of bitches in heat, and stray behavior; the term aggressiveness comprises aggressiveness against other dogs and man (including dominant behavior), either alone or in combination. Beyond the 10 dogs described in this passage, in another three dogs, signs of hypersexuality fully improved with an only partial improvement of the signs of aggressive behavior. In four cases, aggressive behavior did not improve while hypersexuality disappeared. Treatment had no effect on aggressiveness in two dogs, but signs of hypersexuality were partially improved.
